# Inflammation and Gut-Brain Axis During Type 2 Diabetes: Focus on the Crosstalk Between Intestinal Immune Cells and Enteric Nervous System

**DOI:** 10.3389/fnins.2018.00725

**Published:** 2018-10-10

**Authors:** Arnaud Bessac, Patrice D. Cani, Etienne Meunier, Gilles Dietrich, Claude Knauf

**Affiliations:** ^1^NeuroMicrobiota, European Associated Laboratory INSERM/UCLouvain, Brussels, Belgium; ^2^Institut National de la Santé et de la Recherche Médicale, U1220, Université Paul Sabatier, Institut de Recherche en Santé Digestive et Nutrition, Toulouse, France; ^3^Metabolism and Nutrition Research Group, Walloon Excellence in Life Sciences and Biotechnology, Louvain Drug Research Institute, UCLouvain, Université catholique de Louvain, Brussels, Belgium; ^4^Institut de Pharmacologie et de Biologie Structurale, UMR 5089, Université Paul Sabatier, Toulouse, France

**Keywords:** enteric nervous system, immune cells, microbiota, gut-brain axis, diabetes

## Abstract

The gut-brain axis is now considered as a major actor in the control of glycemia. Recent discoveries show that the enteric nervous system (ENS) informs the hypothalamus of the nutritional state in order to control glucose entry in tissues. During type 2 diabetes (T2D), this way of communication is completely disturbed leading to the establishment of hyperglycemia and insulin-resistance. Indeed, the ENS neurons are largely targeted by nutrients (e.g., lipids, peptides) but also by inflammatory factors from different origin (i.e., host cells and gut microbiota). Inflammation, and more particularly in the intestine, contributes to the development of numerous pathologies such as intestinal bowel diseases, Parkinson diseases and T2D. Therefore, targeting the couple ENS/inflammation could represent an attractive therapeutic solution to treat metabolic diseases. In this review, we focus on the role of the crosstalk between intestinal immune cells and ENS neurons in the control of glycemia. In addition, given the growing evidence showing the key role of the gut microbiota in physiology, we will also briefly discuss its potential contribution and role on the immune and neuronal systems.

## Overview of the Different Types of Immune Cells in the Intestine: The Example of its Relation with the Gut Microbiota

The population of gut microbiota is estimated to 100 trillion microorganisms. Thus, our body is composed of 50% of bacteria and 50% of human cells (Cani, 2016). Precisely, the human body encloses approximatively 4.10^13^ bacteria cells with the largest amount inside the large intestine (10^11^ bacteria cells/g of wet stools) (Sender et al., 2016a). In the last estimation, almost 10 million non-redundant microbial genes have been identified in the human gut (Li et al., 2014). The intestinal tract is subjected to an environmental pressure due to the high microbial load of the luminal content (Sender et al., 2016b). Regarding microbial load, one recent article shows that it is the absolute quantity of microbes and not the proportions of microbes that really matters (Vandeputte et al., 2017). Depending of the mode of analysis of gut microbiota population (classical relative abundance-based profiling vs. quantitative microbiome profiling), the interpretation of data could be completely different, and maybe falsely interpreted. The study of gut microbiota impacts in physiology is also complicated due to the controversial data present in the literature. In fact, results could differ depending of numerous factors that we have to take into account such as for example dietary environments, but also the composition and the activity of the gut microbes (Cani, 2018a). To illustrate this controversy, *Prevotella copri* and its metabolites could be considered as pro- or anti-diabetic actions depending on the diet or the model used (Cani, 2018a). Therefore, these few examples clearly highlight the fact that we are still at the beginning of the story, and we will need more time to better understand the gut microbiota and its importance in human health.

Nowadays, the impact of gut microbiota in the control of various physiological functions is proposed (Cani, 2018a). Abnormal composition and/or activity of the gut microbiota are associated with the development of numerous pathologies such as cancer, obesity and type 2 diabetes (T2D) (Cani, 2018a; Cani and Jordan, 2018b; Rastelli et al., 2018). Despite the complexity of the crosstalk, a clear link is established between inflammation and modification of the gut microbiota (Stecher, 2015; Cani, 2018a). Here, we will mainly introduce how gut bacteria could modulate the function of intestinal immune cells, and describe the molecular actors involved.

Intestinal bacteria are physically separated from mucosal immune system by a single epithelial cell layer, which the primary function is to absorb nutrients (small intestine) and water (colon). Mucosal immune system prevent microbial invasion and is tightly regulated. One of its major role is to avoid the development of chronic inflammation and the subsequent loss of the intestinal epithelium integrity. Microfold cells or M cells, in the specialized follicle-associated epithelium overlying Peyer Patches (PP), and isolated lymphoid follicles (ILF) are the major cell types that sample bacteria and associated antigens. Processed bacterial-derived antigens are presented locally (i.e., into the PP or ILF) or within the mesenteric lymph nodes that drain dendritic cells to initiate an adaptive immune response (Wells et al., 2017). Both effector- and regulatory-T lymphocytes scattered within the intestinal mucosa are generated in response to commensal bacterial antigens. At steady state, the number and the spectrum of effector-T lymphocytes subsets that are present within the intestinal mucosa are dependent on the host’s microbiota. Any pathogen invasion, disruptions of the mucus barrier or of the intestinal epithelium integrity, and/or failure in the regulatory mechanisms of the immune response may result in mucosal inflammation (Barreau and Hugot, 2014; Al Nabhani et al., 2017). Immune mediators released upon inflammation are largely dependent on the nature of the microbes triggering the immune system (Maloy and Powrie, 2011). Intestinal epithelial cells and resident innate immune cells sense pathogens locally. The interactions between the pathogens and the pattern-recognition receptors (PRRs) expressed both by stromal and immune cells trigger rapid production of immune and microbicide mediators (e.g., cytokines, chemokines, bioactive lipids, and cell-autonomous immune effectors), which restrict pathogen growth. In parallel, dendritic cells will mature upon contact with microbe-associated molecular patterns (MAMPs) or when some factors are released by injured tissues, namely the damage associated molecular patterns (DAMPs) (i.e., a process allowing antigen presentation to T cells) (Maloy and Powrie, 2011; Geginat et al., 2015). Regarding the intrinsic properties of mature dendritic cells and their soluble (e.g., cytokines, chemokines) and cellular (stromal, myeloid and lymphoid cells) immune environment, antigen-primed CD4^+^ T lymphocytes may acquire different effector functions. Indeed, viral or intracellular bacterial infections drive T lymphocyte commitment toward the Th1 phenotype, a process that relies both on the production of IL-12 and IL-18 by myeloid cells and the subsequent IFNγ released by innate lymphoid cells (ILC)1 (Trinchieri, 2003; Bernink et al., 2013). Th1 CD4^+^ lymphocytes produce high levels of IFNγ but also TNF-α. The clearance of extracellular bacteria and fungi mainly depends on Th17-polarized lymphocytes that produce IL-17, IL-22, IL-21, TNF-α and GMCSF. The differentiation of naïve CD4^+^ T lymphocytes into Th17 involves an environment enriched in TGFβ, IL-1β and STAT3-inducing cytokines including IL-6 and IL-23 produced by myeloid cells (e.g., dendritic cells and macrophages). IL-23 plays a key role by reinforcing Th17 functional activity and inducing production of GMCSF and TNF-α. The immune polarization of ILCs from group 2 by IL-25, IL-33 and thymic stromal lymphopoietin is a process specific to helminth parasites. This leads to a potent IL-13 release, crucial immune mediator of the anti-parasitic response (Neill et al., 2010). This frontline type 2 cytokine signaling drives the differentiation of T lymphocytes toward the Th2 phenotype, which is characterized by the synthesis of IL4, IL-5 and IL-13 cytokines, that are key to restrict helminth infections. In addition to cytokines, CD4^+^ T lymphocytes may also produce neuropeptides including opioids, serotonin and vasoactive intestinal peptide (O’Connell et al., 2006; Boue et al., 2011; Souza-Moreira et al., 2011). Within the site of inflammation, most of these neuropeptides as well as chemokines, proteases and bioactive lipids are also produced by endothelial and epithelial cells, migrant and resident innate immune cells.

How make the link between gut microbiota, immunity and metabolic diseases? The development of metabolic disorders is clearly linked with modification of the gut microbiota population, but there are many other factors in the intestine (e.g., bioactive molecules, metabolites, endocrine cells) that are disturbed in pathological state. The diversity of the gut microbiota may not fully explain the pathologic phenotype. In fact, recent data show the importance of specific alterations that may arise at the level of the crosstalk occurring between gut microbes but also luminal and intestinal factors during metabolic disorders (Aron-Wisnewsky et al., 2018; Cani, 2018). One may also mention the controversy regarding microbial diversity and its potential link with the onset of inflammation. This important topic has been recently reviewed and clearly described the potential interaction between microbes and host cells *via* immune factors (Cani, 2018a). Modifications of the balance (i.e., gut microbiota and immunity) between healthy and pathological situations participate to the establishment of a type 2 diabetic state. For example in this review (Cani, 2018a), it is discussed that the production of the short-chain fatty acids propionate is altered during diabetes. In physiological condition, propionate can bind to GPR-43 located on intestinal lymphocytes. In pathological situations, the decrease of propionate may lead to the lower abundance of specific T cells (mucosal-associated invariant T cells and Treg) in the lamina propria of the gut. Consequently, the gut barrier function could be altered, and some pathogen-associated molecular patterns (PAMPs) can reach the circulation. This is for instance the case for the levels of lipopolysaccharides (LPS) that are significantly increased in the blood, and trigger systemic low-grade inflammation (Cani et al., 2007; Chu et al., 2018) observed in the whole body and can generate the insulin resistance state observed during diabetes (Cani et al., 2012).

## Intestinal Inflammation and Type 2 Diabetes

Obesity and associated metabolic disorders such as T2D are closely related to a « low-grade » inflammation state as described above (Wellen and Hotamisligil, 2005). In addition to well-described tissues (liver, adipose tissue,…) (Hotamisligil, 2006), the intestine may presents an alteration of the gut barrier function thereby leading hyper-permeability, which is associated with a dysbiosis and in some cases with a certain degree of inflammation at the level of the gut (for review see Slyepchenko et al., 2016). Indeed, in response to a high-fat diet (HFD) that induces insulin resistance, it has been proposed that the intestinal tract may develop an inflammatory response characterized by an increase in pro-inflammatory cytokines expression such as TNF-α, IL-1β, IL-6 (Ding et al., 2010; Kim et al., 2012). Some studies have also reported an increase in myeloperoxidase activity (de La Serre et al., 2010) and/or a decrease in IL-18 and IL-22 expression (Everard et al., 2014a; Wang et al., 2014), two cytokines involved in the intestinal barrier homeostasis and the defense against pathogens. However, it is important to note that the degree of inflammation observed during obesity and diabetes does not reach the same magnitude as the one observed during intestinal inflammatory bowel diseases or severe infection. Nevertheless, besides HFD, data also support that the genetic deletion of leptin (ob/ob) or leptin receptors (db/db) in mice leads to the development of obesity and T2D with a significant increase in pro-inflammatory cytokines (TNF-α, IL-1β, IFNγ, IL-6) in the portal vein, as well as an altered gut barrier (Brun et al., 2007). In addition to metabolic consequences, deletion of leptin receptors also alters immunological response against *Entamoeba histolytica* in mice (Guo et al., 2011) as well as in humans (Duggal et al., 2011). The direct link between intestinal inflammation and metabolic disorders is observed in mice knocked-out for TLR5, a component of the innate immune system. TLR5-deficient mice exhibit a diabetic phenotype associated with intestinal inflammation including increase in pro-inflammatory cytokines (IL-1β, TNF-α) and reduction of the length of the colon, one hallmark of intestinal inflammation (Vijay-Kumar et al., 2010; Chassaing et al., 2014).

What are the “local” consequences of this gut inflammation? All mice models above display alterations of the intestinal homeostasis suggesting as close interrelation between “inflammation, microbiota and metabolism” (Geurts et al., 2014). Because of this inflammatory environment, diabetic mice exhibit modifications of intestinal absorptive capacity characterized by an increase of glucose and/or lipids absorption (Ferraris and Vinnakota, 1995; Petit et al., 2007; Mao et al., 2013) that alters the energetic metabolism. Indeed, HFD diabetic mice develop hyperglycemia and hyperinsulinemia (Ding et al., 2010), glucose intolerance and insulin resistance correlated with a decrease in mucus thickness and increased intestinal permeability (Cani et al., 2008; Everard et al., 2013). This is associated with alteration of the gut microbiota composition and activity (Everard et al., 2011, 2014b; Kim et al., 2012; Molinaro et al., 2017) have shown that gut colonization of germ-free mice induces a bi-phasic inflammation that participates to a bi-phasic impairment of glucose metabolism. Interestingly, recent data have also shown in humans a link between gut microbiota and the accumulation of T cells in the gut of obese people following the ingestion of a high-fat diet (Monteiro-Sepulveda et al., 2015). Both the blunted insulin signaling in enterocytes and the altered localization of the glucose transporter GLUT2 in the enterocytes of obese subjects were linked to the nature of cytokines produced by mucosal T cells (Monteiro-Sepulveda et al., 2015). Conversely, in obese and type 2 diabetic patients another study has shown a decreased number of mucosal-associated invariant T cells (MAIT) producing high levels of Th1 and Th17 cytokines (Magalhaes et al., 2015).

Therefore, the molecular partners linking inflammation, gut microbiota and metabolic disorders could be multiple, but the incretin hormones may represent a real potential of interest. The intestinal tract has also the capacity to produce incretins such as Glucagon-Like Peptide-1 (GLP-1) or Gastric Inhibitory Polypeptide (GIP) in response to nutrients and/or bacterial factors (Cani et al., 2013; Rastelli et al., 2018). HFD and ob/ob mice display an alteration of gut endocrine system including the reduction of GIP- and GLP-1-producing cells and a decrease in proglucagon and GIP expression associated with the alteration of incretins secretion in response to an oral glucose load (Morgan et al., 1988; Anini and Brubaker, 2003; Richards et al., 2016; Shimazu-Kuwahara et al., 2017).

In line with these observations, we have demonstrated that prebiotic treatment in diabetic mice restores the level of GLP-1 to improve glucose metabolism (Cani et al., 2006). Moreover, a chronic exposure to TNF-α alters the release of GLP-1 in HFD mice by acting directly on GLP-1-producing cells (Gagnon et al., 2015). Conversely, a 2 weeks treatment with etanercept, an anti-TNF-α drug, reduces hyperglycemia and hyperinsulinemia of HFD mice. Nowadays, this tripartite concept developed in mice is considered as a potential target to treat metabolic disorders (Geurts et al., 2014).

## Gut-Brain Axis and Type 2 Diabetes

The gut-brain axis is shown as a major signaling pathway controlling glucose homeostasis, and its disturbance is associated with a T2D phenotype (Abot et al., 2018a,b). In normal conditions, glucose is detected by intestinal glucose sensors (such as SGLT1, GLUT2, TASR1/2) present on intestinal cells (enteroendocrine and brush cells, enteric glial cells and neurons) which inform the whole body from the presence of glucose *via* the release of various factors (i.e., gut hormones, neurotransmitters, metabolites) (Fournel et al., 2016). Then, intestinal glucose detection generates an afferent nervous message that provokes an increase of hypothalamic nitric oxide (NO) release (Fournel et al., 2017). In turn, this increase of hypothalamic NO release favors the entry of glucose in tissues *via* an activation of the autonomic nervous system (Fournel et al., 2017). In db/db mice, intestinal inflammation perturbs the detection of glucose by enteric glucose sensors (Duparc et al., 2011). In this case, the intestine sends an aberrant nervous message that fails to increase hypothalamic NO release (Duparc et al., 2011). Accordingly, the insulin resistance state observed during T2D is linked to alteration of gut-brain axis. Recently, we have discovered that the enteric nervous system (ENS) is implicated in the maintenance of glycemia in the whole body *via* the brain. One of the main functions of ENS is to regulate contractions of intestinal smooth muscle cells. In physiological conditions, the reduction of intestinal contractions provokes a decreased fed hyperglycemia (Fournel et al., 2017; Abot et al., 2018b). The modifications of mechanical contractions are detected by the hypothalamus, which controls glucose utilization (Fournel et al., 2017). During T2D, intestinal inflammation is clearly associated with a dysfunction of ENS neurons (Gonzalez-Correa et al., 2017), which could favor duodenal hyper-contractility (Abot et al., 2018b). Our group has demonstrated that duodenal hyper-contractility participates to the establishment of hyperglycemia of HFD mice by modulating the hypothalamic NO release (Fournel et al., 2017).

Numerous pathologies that link inflammation, gut microbiota and ENS have been described (Greenwood-Van Meerveld et al., 2017). Along those lines, recent data have shown that specific neurodegenerative diseases (i.e., multiple sclerosis, Parkinson disease) were also characterized by gastrointestinal comorbidities. In these patients, the specific alterations in gut motility and other pre-motor symptoms are linked with changes in the gut microbiota composition and activity (Endres and Schafer, 2018). Whether the couple microbiota and metabolites influencing the intestinal neuronal system may explain the onset of the disease remains to be proven, but it is becoming clear that the ENS can be considered as a cellular target to treat inflammatory diseases (T2D, inflammatory bowel diseases and ulcers).

Therefore, targeting the intestinal inflammation and/or the gut microbiota to restore the glucose and mechano-sensors in type 2 diabetic patients presents a real potential of scientific interest, but remains to be determined.

## How the Inflammation Modulates the Enteric Nervous System?

Environmental changes related to the inflammatory processes that include a large spectrum of innate and adaptive immune responses, may affect ENS including myenteric plexus controlling gut motility (Cani et al., 2006).

Intestinal motility is regulated by ENS that includes intrinsic primary afferent neurons (IPAN), interneurons and choline acetyltransferase (ChAT)-producing excitatory and NO synthase (NOS)-producing inhibitory motor neurons (Abot et al., 2018a). In the context of immune-mediated regulation of intestinal motility, *muscularis* macrophages residing in the myenteric plexus have been largely studied (Mikkelsen, 2010; Muller et al., 2014; De Schepper et al., 2017). However, cytokines, proteases or neuropeptides produced by mucosal immune cells may also modulate the activity of sensory neurons.

IPANs with their axons extending throughout intestinal mucosa and their cell bodies connected with interneurons which synapse with motor neurons may thus constitute central players in the immune-modulation of the intestinal motility. Accordingly, inflammatory cytokines including TNF-α, IL-1β or IL-6 as well as neuromediators released by innate and adaptive immune cells upon intestinal inflammation alter intrinsic and extrinsic enteric sensory neuron activities (Kindt et al., 2010; Basso et al., 2014; Boue et al., 2014; Brierley and Linden, 2014; Buckley et al., 2014; Hughes Moretta et al., 2014; Mawe, 2015). A slower small-bowel transit and reduced smooth muscle contractility have been reported in patients with inflammatory bowel diseases (Rao et al., 1987; Mawe, 2015). In experimental colitis, intestinal motility is either enhanced or reduced depending on the Th1 or Th2 types of inflammation. Th2-driven colitis induces smooth muscle hyper-contractility and augments intestinal transit while Th1-driven colitis is rather associated with hypo-contractility and reduced intestinal transit (Zhao et al., 2003; Shea-Donohue et al., 2012). Moreover, chronic intestinal inflammation often results in increased innervation together with a reduction of the neuronal activation threshold (Villanacci et al., 2008; Brierley and Linden, 2014). The expression of TNF-α receptors (TNFR1, TNFR2) on enteric neurons also suggest that the neuronal activity maybe modulated directly by the inflammatory environment (Chandrasekharan et al., 2013; Gougeon et al., 2013). Actually, TNF-α has the capacity to modulate electrophysiologic properties of neurons *via* a potentiating effect by increasing the action of cholinergic agonists (carbachol) on gut motility (Rehn et al., 2004). This property of TNF-α could explain the negative impact of cytokines on gut motility. In a molecular point of view, the effect of TNF-α is mediated by an upregulation of the Cyclo-Oxygenase (COX-2), which in turn increases the production of an arachidonic acid derived-molecule, Thromboxane A2 (TXA_2_) (Rehn et al., 2005). In experimental colitis, Chandrasekharan et al. (2013) have also demonstrated that TNF-α has an effect on the apoptosis rate of enteric neurons. Treatment with TNF inhibitors provokes a significant decrease of inflammatory markers expression such as inducible NOS (iNOS). IL-1β is another cytokine that has its receptors on ENS neurons (Gougeon et al., 2013). Similarly to TNF-α, IL-1β may increase iNOS mRNA expression in myenteric neurons (Valentine et al., 1996). IL-1β could increase excitability of neurons by directly acting through its receptors expressed on cell surface. Mechanisms of IL-1β including depolarization of the membrane potential, decreased membrane conductance, and increased discharge of action potentials could explain the diarrhea phase, which appears in obese/diabetic patient (Acosta and Camilleri, 2014) or during intestinal inflammation (Xia et al., 1999).

In addition to neurons, the ENS is composed of glial cells that exert a crucial role in intestinal physiology. Suppression of glial cells alters the function of ENS neurons with an increase in excitatory ChAT neurons and a decrease in inhibitory NOS neurons (Aube et al., 2006). Glial cells are exposed to various molecules depending on the environment including immune factors. Proinflammatory cytokines such as IL-1β and/or low IL-10 concentrations have the capacity to block glial cells proliferation (Ruhl et al., 2001a), highlighting links between immune system and enteric nervous system. Moreover, IL-1β has also the capacity to regulate secretory functions of glial cells by increasing IL-6 synthesis and secretion (Ruhl et al., 2001b). In turn, IL-6 may modulate T cell apoptosis or control gut motility to delay gastric emptying (Atreya et al., 2000; Lang Lehrskov et al., 2018).

## Summary/Conclusion

**FIGURE 1:**
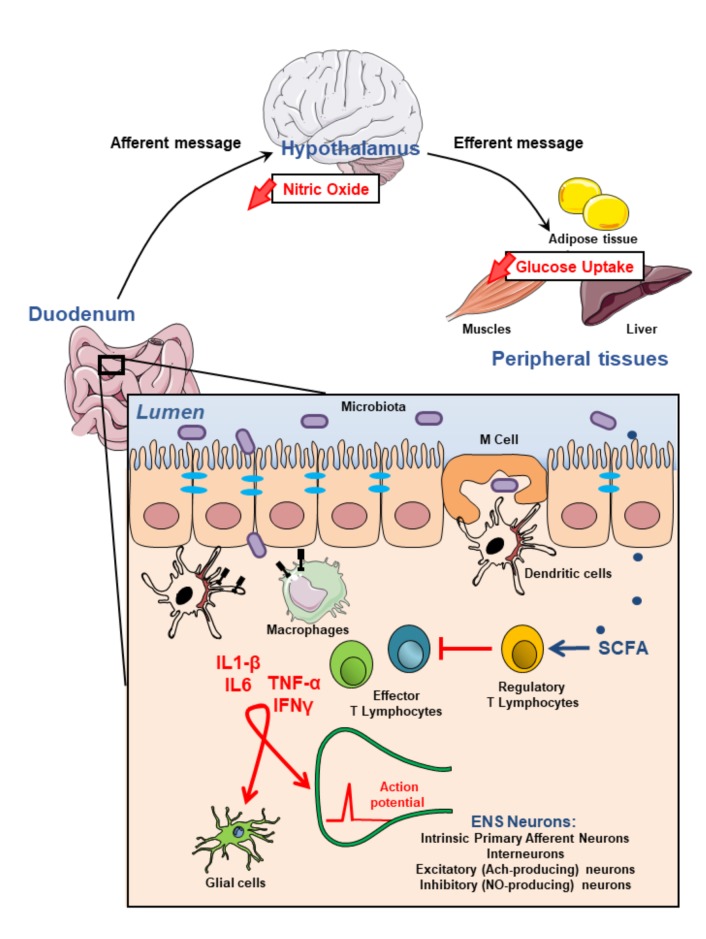
Modulation of the enteric nervous system activity by intestinal immune system may impact on glycemia regulation. Most microbiota-derived products activate both epithelial cells and innate immune cells through pathogen recognition receptors such as toll-like receptors. They also initiate, mainly *via* microbe sampling through M(icrofold) cells, an adaptive immune response. This physiological immune response against intestinal flora generating pro-inflammatory effector T lymhocytes is tighly regulated by regulatory T lymphocytes which induction is associated with the production of short chain fatty acids (SCFAs) by commensal bacteria. Failure of the immune homeostasis may result in the release of higher amounts of proinflammatory cytokines including TNFα, IL1-β, IL6, IFNγ which, in turn, modulate either directly or through glial cells, enteric nervous system (ENS) activity. These alterations of the ENS contribute to impair gut-brain-peripheral axis leading to hyperglycemia and insulin-resistance.

T2D is associated with modification of the gut physiology including alterations of the function of immune cells and ENS. The crosstalk between the two partners is under the influence of the gut bacteria, which release bioactive factors that could participate as intermediate signaling molecules. Some evidence suggests that the establishment of T2D phenotypes (i.e., hyperglycemia, insulin resistance) may have a potential enteric origin; this is also the case of some neurological disorders (e.g., multiple sclerosis, Parkinson disease, autism). Deciphering the impact of “immune cells-ENS” communication and its impact on “gut-brain” axis could represent a new therapeutic perspective for diabetic patients (**Figure 1**). Therapeutic strategies have to take into account the importance of intestinal inflammation and its consequence on integrity of ENS. The gut microbiota and its bioactive releasing factors could represent one potential solution.

## Author Contributions

AB, PDC, EM, GD, and CK have wrote the manuscript.

## Conflict of Interest Statement

The authors declare that the research was conducted in the absence of any commercial or financial relationships that could be construed as a potential conflict of interest.
